# Stereopure ASOs: An unanticipated increase in selectivity for targeting mutant HTT

**DOI:** 10.1016/j.omtn.2024.102312

**Published:** 2024-08-28

**Authors:** Vijay N. Gulumkar, Steven F. Dowdy

**Affiliations:** 1 Department of Cellular & Molecular Medicine, University of California, San Diego, School of Medicine, La Jolla, CA, USA

## Main text

Huntington’s disease (HD) is a neurological genetic disorder caused by the expansion of cytosine-adenine-guanine (CAG) triplet repeats in the wild type of the Huntingtin gene (WT-HTT). The accumulation of more than 36 CAG repeats in WT-HTT leads to the formation of mutant Huntingtin (mHTT), leading to a toxic gain of function.^1^ In HD, mHTT protein accumulates in the brain and drives protein aggregation, transcriptional dysregulation, and dominant-negative interference with WT-HTT protein activity.^2^^,^^3^ While there are currently no approved disease-modifying therapies available, therapeutic approaches that reduce both WT-HTT and mHTT proteins are currently in clinical trials. Although preclinical studies indicate that a reduction of approximately 45% in WT-HTT protein can be tolerated by healthy animals (without mHTT and HD), the safety of long-term reduction of WT-HTT in patients with HD remains uncertain.^4^ Indeed, multiple studies have shown strong evidence that WT-HTT is neuroprotective during stress, especially in HD models.^5^^,^^6^ Thus, given this uncertainty, selectively reducing mtHTT, while leaving WT-HTT levels unchanged, is the preferred therapeutic approach in HD.

Based on their Watson-Crick base-pairing mechanism of action, antisense oligonucleotide (ASO) and small interfering RNA (siRNA) therapeutics have great potential to treat HD.^7^ Indeed, both ASOs and siRNAs that target the CAG triplet repeats have shown promise in reducing HTT levels.^8^^,^^9^ However, a more selective approach of targeting single-nucleotide polymorphisms (SNPs) associated with mHTT shows greater promise of differentiating between mHTT and WT-HTT.^10^ Bilsen et al. showed that selectively targeting an HD-associated SNP several thousand bases downstream of the CAG repeat with an siRNA selectively lowered the pathogenic allele mRNA by 80%.^8^ Likewise, Carroll et al. developed an ASO to SNP sites in HTT that selectively silenced mHTT.^9^ These allele-specific approaches enable the rapid selection of oligonucleotide therapeutics for patients with HD based on potency, selectivity, and population coverage.

ASOs typical have a full (or nearly full) phosphorothioate (PS) backbone, where one of the non-bridging oxygen atoms is replaced with a more “hydrophobic” sulfur atom that results in both a significant increase in metabolic stability and protein binding and an enhanced escape from endosomes.^1^^,^^2^^,^^4^ However, during synthesis, the sulfur atom is randomly placed in either the Sp or Rp stereospecific position at each internucleotide linkage, resulting in a stereomixmer of >10^5^ stereoisomers.^11^ Currently, there are 7 approved stereomixmer ASOs, so clearly most, if not all, of these stereoisomers are functionally active to one extent or another. However, for more than 10 years, there has been an ongoing debate on the benefits of stereomixmer vs. stereopure ASOs^12^ (and this commentary is not going to resolve that argument in <600 words!).

Here, Iwamoto et al. from Wave Life Science in Cambridge, MA, adds to that debate by synthesizing a stereopure gapmer ASO (WVE-003) targeting SNP3 (rs362273) for allele-selective reduction of mHTT.^13^ The WVE-003 ASO also contains three stereospecific phosphoryl guanidine (PN) internucleotide linkages in the gapmer’s RNA wings. Interestingly, the results here incorporating stereopure PN modifications showed improved allele selectivity over prior molecules HTT-164 (WVE-120101) and HTT-273 (WVE-120102), which were withdrawn from the clinics due to their failure to consistently reduce mHTT levels in the cerebrospinal fluid (CSF) of patients with HD. Thus, incorporating PN modification into SNP3-targeting molecules improves their overall activity profiles. Impressively, the WVE-003 ASO showed a strong SNP3 allele-specific mHTT knockdown vs. WT-HTT or other SNP-containing mHTT alleles. Together, the stereospecific PS and PN modifications resulted in a substantial increase in selectivity, potency, durability, and delivery into cells in culture and the CNS of preclinical mouse models.

WVE-003 is also currently being evaluated in clinical trials (ClinicalTrials.gov: NCT05032196).^14^ As of this writing, the primary findings from the SELECT-HD study indicate that WVE-003 may reduce mHTT in patients while retaining WT-HTT activity. Therefore, an allele-selective approach that spares WT-HTT while maintaining its neuroprotective effects may be the most effective way to deliver a disease-modifying HD treatment.

## Declaration of interests

The authors declare no competing interests.
